# Neuronal PPP2R5C in plasma is a potential biomarker for early diagnosis of Alzheimer's disease

**DOI:** 10.1002/alz70856_101828

**Published:** 2025-12-25

**Authors:** Shilin Luo, Hui Liu, Tingting Xiao, Zhentao Zhang, Bin Jiao, Lu Shen

**Affiliations:** ^1^ Xiangya Hospital, Central South University, Changsha, Hunan, China; ^2^ Renmin Hospital of Wuhan University, Wuhan, China

## Abstract

**Background:**

Overwhelming studies have demonstrated that implementing an early intervention is the most effective strategy to impede the advancement of Alzheimer's disease (AD), depending on the identification of early diagnostic biomarkers.

**Method:**

In this study, we first employed proteomics to identify differentially expressed proteins in neuron‐derived exosomes (NDEs) from plasma samples of 13 familial AD (FAD) patients, 10 pre‐FAD patients, and 18 cognitively normal (CN) controls. These findings were subsequently validated using targeted mass spectrometry in a cohort of sporadic AD individuals. Enzyme‐linked immunosorbent assay (ELISA) was utilized for further validation in a larger cohort comprising 74 AD patients, 76 individuals with amnestic mild cognitive impairment (aMCI), and 74 healthy controls. Additionally, the diagnostic performance of PPP2R5C was evaluated by analyzing 34 AD patients, 28 progressive supranuclear palsy (PSP) patients, and 37 frontotemporal dementia (FTD) patients. Meanwhile, the mechanism underlying PPP2R5C's role in AD pathogenesis was explored through in *vitro* and in *vivo* models.

**Result:**

Our findings reveal a trend of decreased PPP2R5C protein levels in NDEs across FAD, pre‐FAD, and CN (FAD < pre‐FAD < CN), as well as a similar downward trend in plasma PPP2R5C levels within the AD cohort (AD < aMCI < CN, *p* <0.05). Diagnostic accuracy was assessed using receiver operating characteristic (ROC) curve analysis. Plasma PPP2R5C demonstrated significant diagnostic ability for AD (AUC = 0.84, *p* < 0.0001) and for aMCI (AUC = 0.74, *p* < 0.0001). Mechanistically, we found that PPP2R5C interacts with Tau and attenuates both its total and phosphorylated levels, either by mediating the autophagolysosomal pathway or directly influencing PP2A activity. Furthermore, PPP2R5C‐mediated autophagy was shown to be dependent on its binding activity to unc‐51‐like kinase 1 (ULK1).

**Conclusion:**

This study suggested that plasma PPP2R5C could be a novel and ideal biomarker for the early diagnosis of AD and its involvement in the pathogenesis of AD

